# Supplementary material for: Network hubs in root-associated fungal metacommunities
Source: Microbiome. 2018 Jun 23;6:116. doi: 10.1186/s40168-018-0497-1 (PMC6015470; doi:10.1186/s40168-018-0497-1)
Supplement: Supplementary file 6 — Figure S1. Number of sequencing reads, interaction specificity, and local betweenness. (DOCX 428 kb) [file 40168_2018_497_MOESM6_ESM.docx]

**
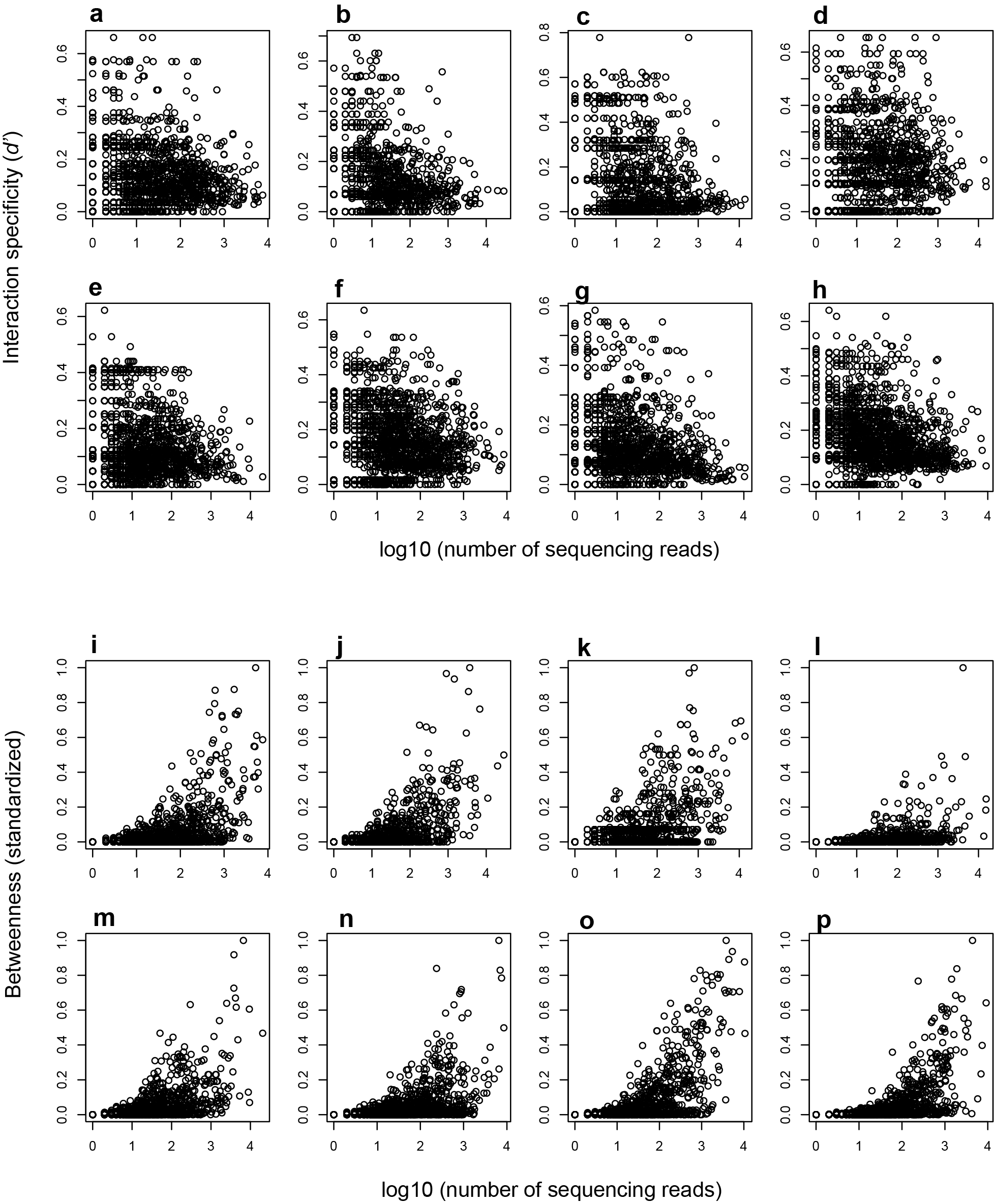
**

**Additional file 6; Figure S1.** Number of sequencing reads, interaction specificity, and local betweenness. (**a-h**) Relationship between the number of sequencing reads and interaction specificity is shown for each local forest (**a**, site 1; **b**, site 2; **c**, site 3; **d**, site, 4; **e**, site 5; **f**, site 6; **g**, site 7; **h**, site 8). (**i-p**) Relationship between the number of sequencing reads and local betweenness is shown for each local forest (**i**, site 1; **j**, site 2; **k**, site 3; **l**, site, 4; **m**, site 5; **n**, site 6; **o**, site 7; **p**, site 8).
