# Supplementary material for: Network hubs in root-associated fungal metacommunities
Source: Microbiome. 2018 Jun 23;6:116. doi: 10.1186/s40168-018-0497-1 (PMC6015470; doi:10.1186/s40168-018-0497-1)
Supplement: Supplementary file 7 — Figure S2. Structure of plant–fungus networks in each local forest. (DOCX 4270 kb) [file 40168_2018_497_MOESM7_ESM.docx]

**
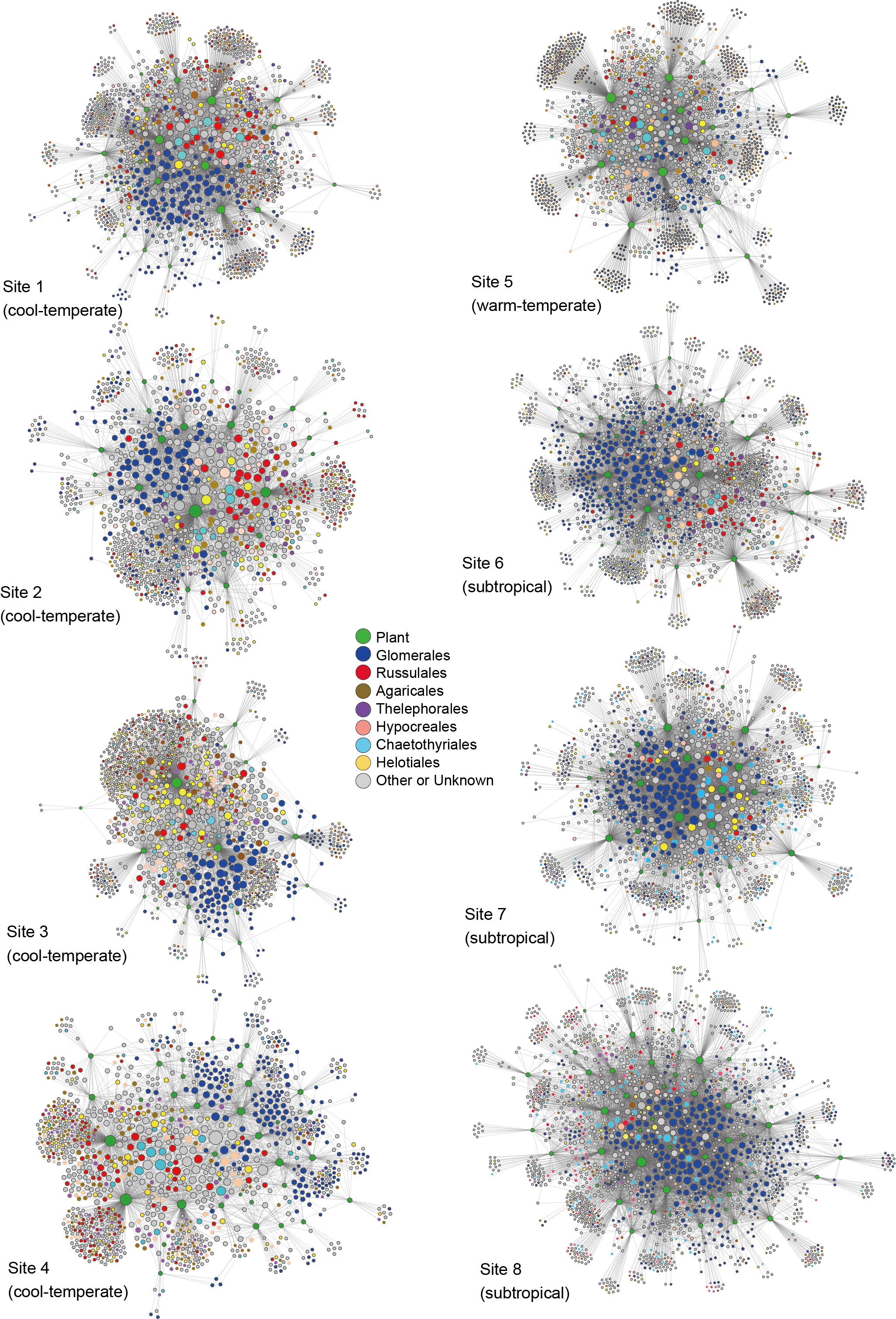
**

**Additional file 7; Figure S2.** Structure of plant–fungus networks in each local forest. The order-level taxonomy of constituent fungal OTUs is indicated by color.
