# Supplementary material for: Network hubs in root-associated fungal metacommunities
Source: Microbiome. 2018 Jun 23;6:116. doi: 10.1186/s40168-018-0497-1 (PMC6015470; doi:10.1186/s40168-018-0497-1)
Supplement: Supplementary file 8 — Figure S3. Locality information within the full metacommunity-scale network. (DOCX 4949 kb) [file 40168_2018_497_MOESM8_ESM.docx]

**
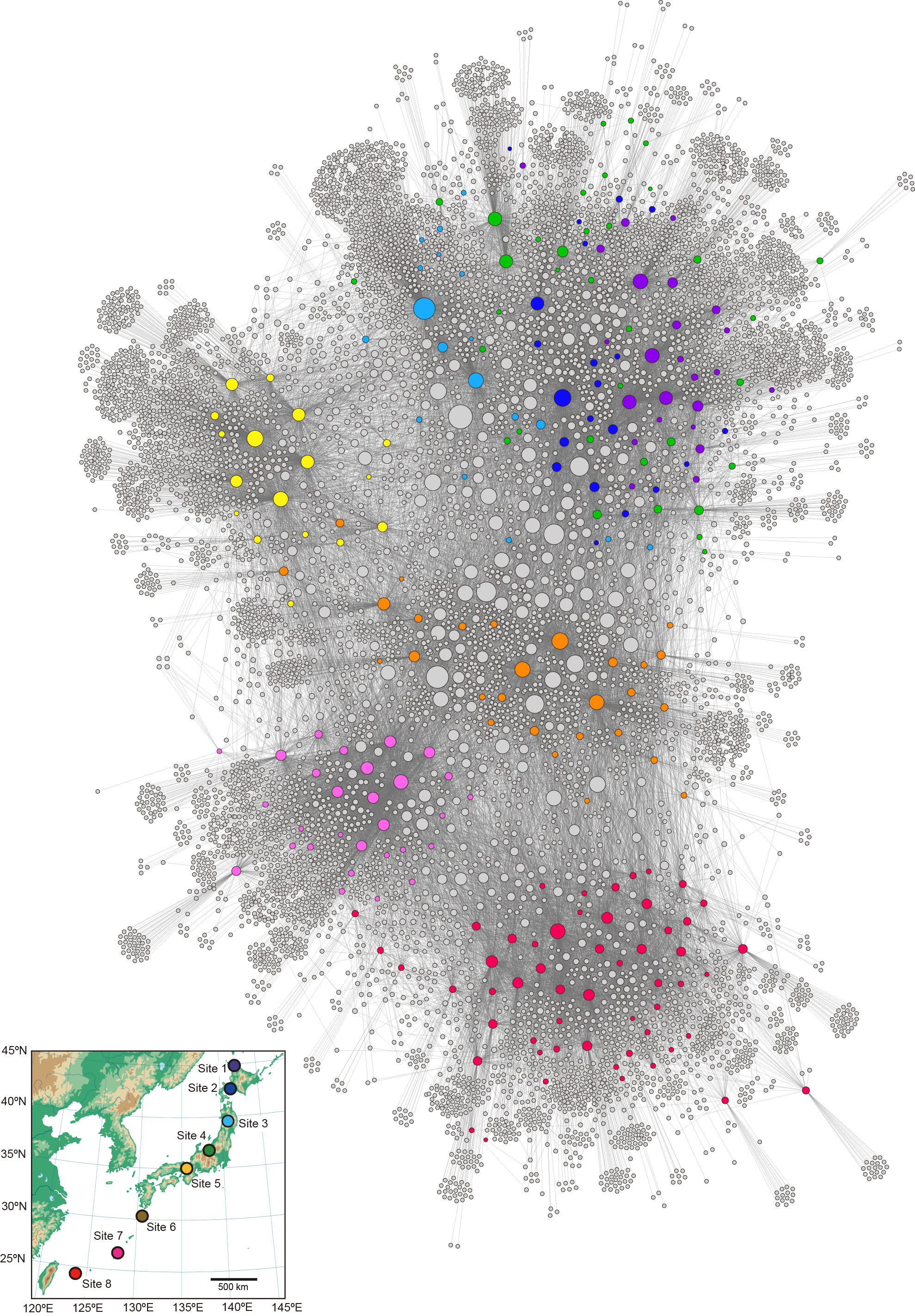
**

**Additional file 8; Figure S3.** Locality information within the full metacommunity-scale network. Plant species/taxa observed in each local forest are indicated by the color series defined in the map. All fungal OTUs are indicated by grey.
