# Supplementary material for: Network hubs in root-associated fungal metacommunities
Source: Microbiome. 2018 Jun 23;6:116. doi: 10.1186/s40168-018-0497-1 (PMC6015470; doi:10.1186/s40168-018-0497-1)
Supplement: Supplementary file 9 — Figure S4. Metacommunity-scale network of cool-temperate forests. (DOCX 3939 kb) [file 40168_2018_497_MOESM9_ESM.docx]

**
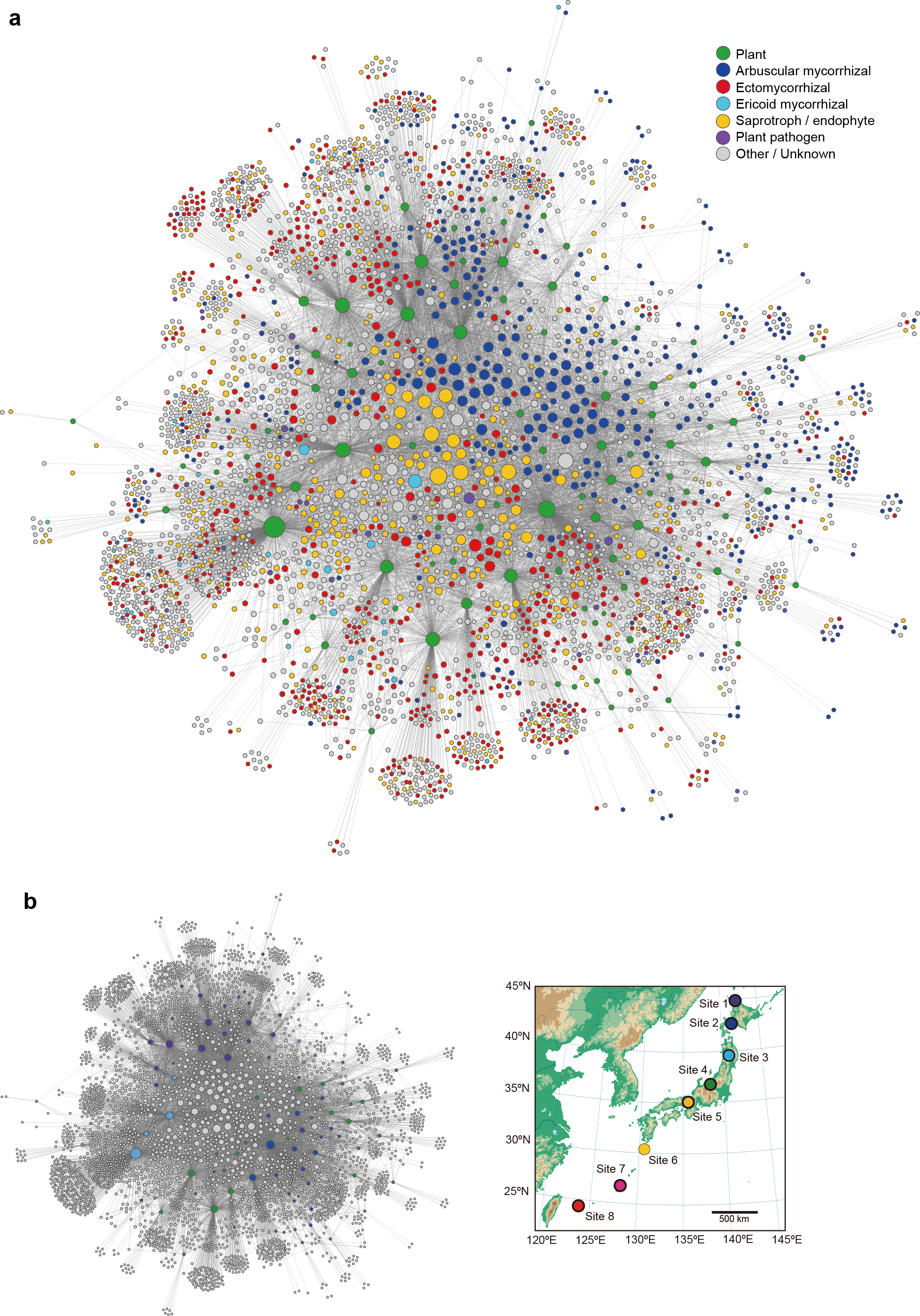
**

**Additional file 9; Figure S4.** Metacommunity-scale network of cool-temperate forests. The local networks of the four cool-temperate forests (sites 1–4; Additional file 5; Figure S2) were merged into a metacommunity-scale network. **a**, Functional groups of fungi within the metacommunity network. **b**, Locality information. Plant species/taxa observed in each local forest are indicated by the color series defined in the map. All fungal OTUs are indicated by grey.
