# Supplementary material for: Network hubs in root-associated fungal metacommunities
Source: Microbiome. 2018 Jun 23;6:116. doi: 10.1186/s40168-018-0497-1 (PMC6015470; doi:10.1186/s40168-018-0497-1)
Supplement: Supplementary file 10 — Figure S5. Metacommunity-scale network of warm-temperate and subtropical forests. (DOCX 4792 kb) [file 40168_2018_497_MOESM10_ESM.docx]

**
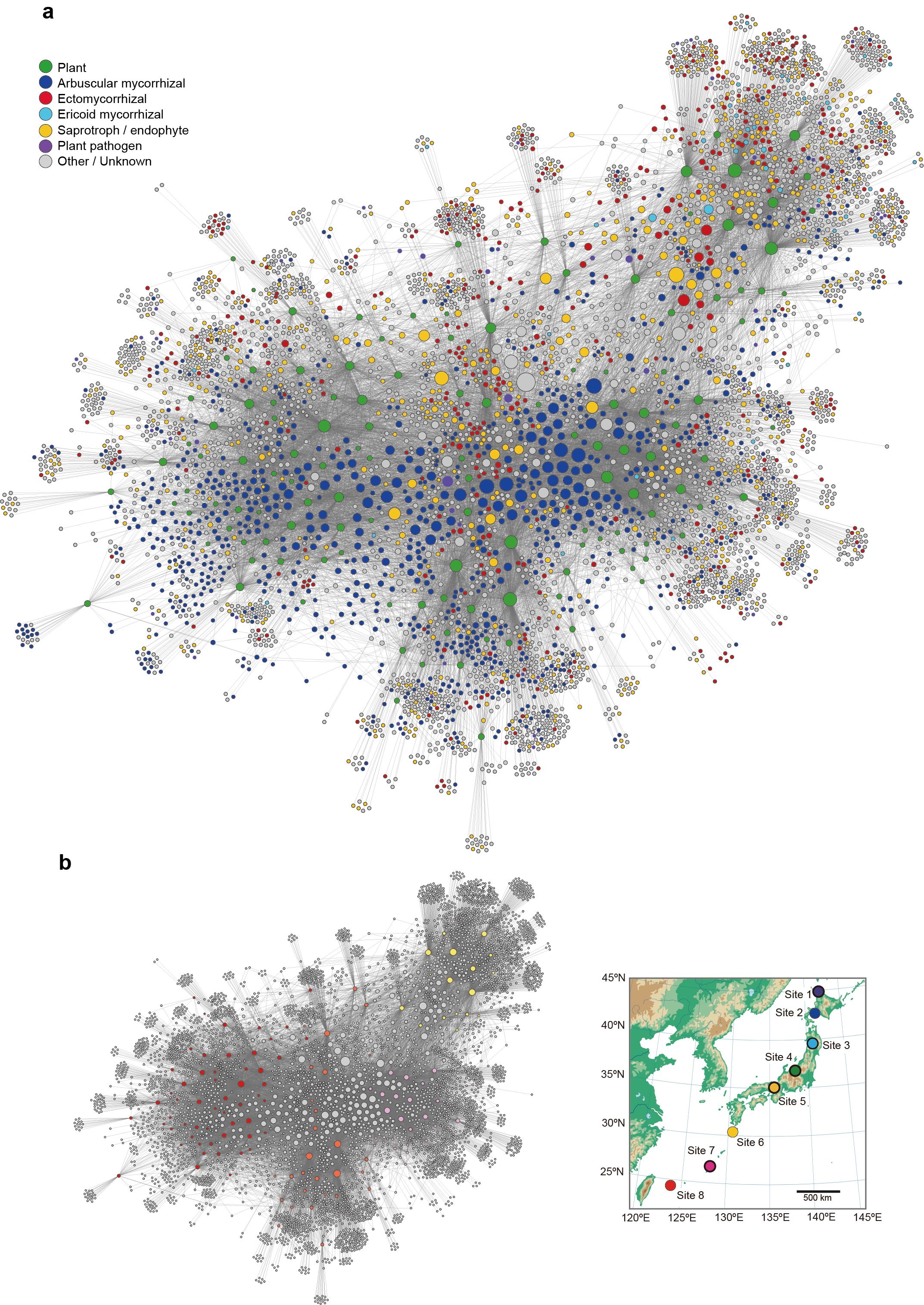
**

**Additional file 10; Figure S5.** Metacommunity-scale network of warm-temperate and subtropical forests. The local networks of the warm-temperate forest and the three subtropical forests (sites5–8; Additional file 5; Figure S2) were merged into a metacommunity-scale network. **a**, Functional groups of fungi within the metacommunity network. **b**, Locality information. Plant species/taxa observed in each local forest are indicated by the color series defined in the map. All fungal OTUs are indicated by grey.
