# Supplementary material for: Network hubs in root-associated fungal metacommunities
Source: Microbiome. 2018 Jun 23;6:116. doi: 10.1186/s40168-018-0497-1 (PMC6015470; doi:10.1186/s40168-018-0497-1)
Supplement: Supplementary file 11 — Table S1. Top 10 list of non-Glomeromycota OTUs with the highest betweenness within the subtropical metacommunity network. (DOCX 114 kb) [file 40168_2018_497_MOESM11_ESM.docx]

**Additional file 11; Table S1.** Top-10 list of non-Glomeromycota OTUs with highest betweenness within the subtropical metacommunity network. In the metacommunity-scale network consisting of three subtropical forests (sites 6–8), fungal OTUs were ranked based on their betweenness centrality scores. As taxonomic information of Glomeromycota OTUs with high betweenness scores was redundant (e.g., *Glomus* spp. or Glomeraceae spp.), the top-10 list of non-Glomeromycota OTUs are shown. Taxonomy information of each OTU was inferred based on the query-centric auto-*k*-nearest-neighbor algorithm of reference database search [57, 58] and subsequent taxonomic assignment with the lowest common ancestor algorithm [59]. The results of the NCBI nucleotide Blast are also shown. For simplicity, the functional groups of fungi inferred with the program FUNGuild [60] were organized into several categories

| OTU | Score | Phylum | Class | Order | Family | Genus | Category | OTU | NCBI top hit | Accession | Cover | Identity |
| --- | --- | --- | --- | --- | --- | --- | --- | --- | --- | --- | --- | --- |
| F_0381 | 1.000 | Basidiomycota | Tremellomycetes | Trichosporonales | Trichosporonaceae | *Cryptococcus* | Others_Unknown | F_0381 | *Saitozyma podzolica** | KY320605 | 92% | 99% |
| F_0073 | 0.662 | Ascomycota | Sordariomycetes | - | - | *-* | Others_Unknown | F_0073 | *Rhexodenticula acaciae* | KY173442 | 94% | 95% |
| F_0007 | 0.633 | Ascomycota | Sordariomycetes | Diaporthales | Melanconidaceae | *Melanconiella* | Saprotroph/Endophyte | F_0007 | *Melanconiella elegans* | KJ173701 | 100% | 85% |
| F_0510 | 0.511 | Ascomycota | Sordariomycetes | Hypocreales | Nectriaceae | *Calonectria* | Plant_Pathogen | F_0510 | *Calonectria ilicicola* | JQ809270 | 100% | 100% |
| F_0610 | 0.507 | Ascomycota | Sordariomycetes | Hypocreales | Hypocreaceae | *Trichoderma* | Saprotroph/Endophyte | F_0610 | *Trichoderma spirale* | KU948158 | 100% | 100% |
| F_0079 | 0.506 | Ascomycota | Sordariomycetes | Hypocreales | Nectriaceae | *-* | Saprotroph/Endophyte | F_0079 | *Ilyonectria protearum* | NR_152890 | 99% | 100% |
| F_0009 | 0.498 | Ascomycota | Sordariomycetes | Diaporthales | Melanconidaceae | *Melanconiella* | Saprotroph/Endophyte | F_0009 | *Melanconiella elegans* | KJ173701 | 100% | 85% |
| F_1188 | 0.463 | Basidiomycota | Tremellomycetes | Trichosporonales | Trichosporonaceae | *Cryptococcus* | Others_Unknown | F_1188 | *Saitozyma podzolica** | KY320605 | 92% | 99% |
| F_0042 | 0.452 | - | - | Mortierellales | Mortierellaceae | *Mortierella* | Saprotroph/Endophyte | F_0042 | *Mortierella humilis* | KP714537 | 100% | 100% |
| F_0035 | 0.404 | Ascomycota | - | - | - | *-* | Others_Unknown | F_0035 | Sordariomycetes sp. | KY591297 | 84% | 100% |

***Synonym, *Cryptcoccus podzolica*
