# Supplementary material for: Network hubs in root-associated fungal metacommunities
Source: Microbiome. 2018 Jun 23;6:116. doi: 10.1186/s40168-018-0497-1 (PMC6015470; doi:10.1186/s40168-018-0497-1)
Supplement: Supplementary file 12 — Table S2. Top 10 list of non-Glomeromycota OTUs with the highest weighted betweenness within the metacommunity networks. (DOCX 125 kb) [file 40168_2018_497_MOESM12_ESM.docx]

**Additional file 12: Table S2.** Top-10 list of non-Glomeromycota OTUs with the highest weighted betweenness within the metacommunity networks. In each of the three metacommunity-scale networks examined (full, cool-temperate, and warm-temperate/subtropical), fungal OTUs were ranked based on their weighted betweenness centrality scores. As taxonomic information of Glomeromycota OTUs with high betweenness scores was redundant (e.g., *Glomus* spp. or Glomeraceae spp.), the top-10 list of non-Glomeromycota OTUs is shown. Taxonomy information of each OTU was inferred based on the query-centric auto-*k*-nearest-neighbor algorithm of reference database search and subsequent taxonomic assignment with the lowest common ancestor algorithm. The results of the NCBI nucleotide Blast are also shown. For simplicity, the functional groups of fungi inferred with the program FUNGuild were organized into several categories. See Data S4 (Additional file 4) for details of the categories and for full results including Glomeromycota and other fungal OTUs.

| OTU | Score | Phylum | Class | | Order | | | Family | | | | Genus | | | | Category | | | | | NCBI Blast top hit | | | | | | | Accession | | Cover | | Identity |
| --- | --- | --- | --- | --- | --- | --- | --- | --- | --- | --- | --- | --- | --- | --- | --- | --- | --- | --- | --- | --- | --- | --- | --- | --- | --- | --- | --- | --- | --- | --- | --- | --- |
| Full (8 sites) | | | |  | | |  | | |  | | |  | | | |  | | | | | | |  | | |  | |  | |  |  |
| F_0756 | 1.000 | Ascomycota | Sordariomycetes | | - | | | - | | | | *-* | | | | Others_Unknown | | | | | *Phialemoniopsis curvata* | | | | | | | AB278180 | | 100% | | 100% |
| F_0368 | 0.959 | Basidiomycota | Malasseziomycetes | | Malasseziales | | | Malasseziaceae | | | | *Malassezia* | | | | Others_Unknown | | | | | *Malassezia restricta* | | | | | | | KT809059 | | 100% | | 100% |
| F_0381 | 0.930 | Basidiomycota | Tremellomycetes | | Trichosporonales | | | Trichosporonaceae | | | | *Cryptococcus* | | | | Others_Unknown | | | | | *Saitozyma podzolica** | | | | | | | KY320605 | | 92% | | 99% |
| F_0489 | 0.795 | - | - | | Mortierellales | | | Mortierellaceae | | | | *Mortierella* | | | | Saprotroph/Endophyte | | | | | *Mortierella* sp. | | | | | | | KM113754 | | 100% | | 100% |
| F_1968 | 0.780 | Ascomycota | Sordariomycetes | | Xylariales | | | Sporocadaceae | | | | *Pestalotiopsis* | | | | Plant_Pathogen | | | | | *Pestalotiopsis disseminata* | | | | | | | AB251918 | | 100% | | 100% |
| F_0181 | 0.773 | Ascomycota | Leotiomycetes | | Helotiales | | | Dermateaceae | | | | *Pezicula* | | | | Saprotroph/Endophyte | | | | | *Pezicula melanigena* | | | | | | | LC206665 | | 100% | | 99% |
| F_0079 | 0.732 | Ascomycota | Sordariomycetes | | Hypocreales | | | Nectriaceae | | | | *-* | | | | Saprotroph/Endophyte | | | | | *Ilyonectria protearum* | | | | | | | NR_152890 | | 99% | | 100% |
| F_1188 | 0.649 | Basidiomycota | Tremellomycetes | | Trichosporonales | | | Trichosporonaceae | | | | *Cryptococcus* | | | | Others_Unknown | | | | | *Saitozyma podzolica** | | | | | | | KY320605 | | 92% | | 99% |
| F_0610 | 0.566 | Ascomycota | Sordariomycetes | | Hypocreales | | | Hypocreaceae | | | | *Trichoderma* | | | | Saprotroph/Endophyte | | | | | *Trichoderma spirale* | | | | | | | KU948158 | | 100% | | 100% |
| F_0764 | 0.559 | Ascomycota | Eurotiomycetes | | Chaetothyriales | | | Herpotrichiellaceae | | | | *Cladophialophora* | | | | Saprotroph/Endophyte | | | | | *Cladophialophora* sp. | | | | | | | AB986416 | | 92% | | 100% |
|  |  |  |  | |  | | |  | | | |  | | | |  | | | | |  | | | | | | |  | |  | |  |
| Northern 4 sites (cool-temperate) | | | | | |  | | |  | | | | |  | | | | |  | | | |  | |  |  |  |  |  |  |  |  |
| F_0368 | 1.000 | Basidiomycota | Malasseziomycetes | | Malasseziales | | | Malasseziaceae | | | | *Malassezia* | | | | Others_Unknown | | | | | *Malassezia restricta* | | | | | | | KT809059 | | 100% | | 100% |
| F_0181 | 0.725 | Ascomycota | Leotiomycetes | | Helotiales | | | Dermateaceae | | | | *Pezicula* | | | | Saprotroph/Endophyte | | | | | *Pezicula melanigena* | | | | | | | LC206665 | | 100% | | 99% |
| F_0756 | 0.630 | Ascomycota | Sordariomycetes | | - | | | - | | | | *-* | | | | Others_Unknown | | | | | *Phialemoniopsis curvata* | | | | | | | AB278180 | | 100% | | 100% |
| F_0768 | 0.613 | Ascomycota | Sordariomycetes | | Hypocreales | | | Nectriaceae | | | | *-* | | | | Saprotroph/Endophyte | | | | | *Neonectria obtusispora* | | | | | | | LC206668 | | 99% | | 100% |
| F_1770 | 0.601 | Ascomycota | Sordariomycetes | | Hypocreales | | | Hypocreaceae | | | | *Trichoderma* | | | | Saprotroph/Endophyte | | | | | *Trichoderma virens* | | | | | | | KY950294 | | 98% | | 99% |
| F_0781 | 0.586 | - | - | | Mortierellales | | | Mortierellaceae | | | | *Mortierella* | | | | Saprotroph/Endophyte | | | | | *Mortierella* sp. | | | | | | | JQ341136 | | 100% | | 99% |
| F_0079 | 0.548 | Ascomycota | Sordariomycetes | | Hypocreales | | | Nectriaceae | | | | *-* | | | | Saprotroph/Endophyte | | | | | *Ilyonectria protearum* | | | | | | | NR_152890 | | 99% | | 100% |
| F_0153 | 0.533 | Ascomycota | - | | - | | | - | | | | *Tetracladium* | | | | Saprotroph/Endophyte | | | | | *Tetracladium furcatum* | | | | | | | KY322519 | | 100% | | 99% |
| F_1997 | 0.506 | Ascomycota | Sordariomycetes | | Sordariales | | | Chaetomiaceae | | | | *-* | | | | Others_Unknown | | | | | *Chaetomium* sp. | | | | | | | KU059823 | | 100% | | 99% |
| F_0001 | 0.500 | Basidiomycota | Agaricomycetes | | Russulales | | | Russulaceae | | | | *Lactarius* | | | | Ectomycorrhizal | | | | | *Lactarius quietus* | | | | | | | KT165231 | | 100% | | 97% |
|  |  |  |  | |  | | |  | | | |  | | | |  | | | | |  | | | | | | |  | |  | |  |
| Southern 4 sites (warm-temperate and subtropical) | | | | | | |  | | | |  | | | |  | | |  | |  | |  |  |  |  |  |  |  |  |  |  |  |
| F_0610 | 1.000 | Ascomycota | Sordariomycetes | | Hypocreales | | | Hypocreaceae | | | | *Trichoderma* | | | | Saprotroph/Endophyte | | | | | *Trichoderma spirale* | | | | | | | KU948158 | | 100% | | 100% |
| F_1188 | 0.534 | Basidiomycota | Tremellomycetes | | Trichosporonales | | | Trichosporonaceae | | | | *Cryptococcus* | | | | Others_Unknown | | | | | *Saitozyma podzolica** | | | | | | | KY320605 | | 92% | | 99% |
| F_0756 | 0.475 | Ascomycota | Sordariomycetes | | - | | | - | | | | *-* | | | | Others_Unknown | | | | | *Phialemoniopsis curvata* | | | | | | | AB278180 | | 100% | | 100% |
| F_0017 | 0.462 | Ascomycota | - | | - | | | - | | | | *-* | | | | Others_Unknown | | | | | *Scleropezicula* sp. | | | | | | | KT809119 | | 100% | | 98% |
| F_1968 | 0.455 | Ascomycota | Sordariomycetes | | Xylariales | | | Sporocadaceae | | | | *Pestalotiopsis* | | | | Plant_Pathogen | | | | | *Pestalotiopsis disseminata* | | | | | | | AB251918 | | 100% | | 100% |
| F_0381 | 0.455 | Basidiomycota | Tremellomycetes | | Trichosporonales | | | Trichosporonaceae | | | | *Cryptococcus* | | | | Others_Unknown | | | | | *Saitozyma podzolica** | | | | | | | KY320605 | | 92% | | 99% |
| F_1839 | 0.438 | - | - | | Mortierellales | | | Mortierellaceae | | | | *Mortierella* | | | | Saprotroph/Endophyte | | | | | *Mortierella elongata* | | | | | | | MH047197 | | 100% | | 97% |
| F_0489 | 0.416 | - | - | | Mortierellales | | | Mortierellaceae | | | | *Mortierella* | | | | Saprotroph/Endophyte | | | | | *Mortierella* sp. | | | | | | | KM113754 | | 100% | | 100% |
| F_1728 | 0.400 | Basidiomycota | - | | - | | | - | | | | *-* | | | | Others_Unknown | | | | | *Sakaguchia lamellibrachiae* | | | | | | | AB263120 | | 100% | | 88% |
| F_1778 | 0.375 | Basidiomycota | Tremellomycetes | | Trichosporonales | | | Trichosporonaceae | | | | *Cryptococcus* | | | | Others_Unknown | | | | | *Saitozyma podzolica** | | | | | | | KY320605 | | 91% | | 99% |

*Synonym, *Cryptcoccus podzolica*
